# Experiences of surgical complications and reoperations in nonsyndromic sagittal synostosis patients in Oulu

**DOI:** 10.1007/s00381-024-06519-0

**Published:** 2024-06-28

**Authors:** Anja Svalina, Willy Serlo, Juha-Jaakko Sinikumpu, Niina Salokorpi

**Affiliations:** 1 Research Unit of Clinical Medicine, Oulu University Hospital and University of Oulu, Oulu, Finland; 2 Medical Research Center, Oulu University Hospital, Oulu, Finland; 3 Skills Center for Children and Women, Oulu University Hospital, Oulu, Finland; 4 Department of Neurosurgery, NeurocenterOulu University Hospital, PO Box 21, 90029 Oulu, OYS Finland

**Keywords:** Scaphocephaly, Craniocerebral disproportion, Comorbidity, Aesthetical result

## Abstract

**Objective:**

The purpose of this study was to evaluate the surgical complications of patients treated for nonsyndromic sagittal craniosynostosis and the necessity for reoperations due to craniocerebral disproportion.

**Materials and methods:**

The patient cohort of this study consisted of patients (*N* = 82) who were treated in the Oulu University Hospital using the open vault cranioplasty with a modified H-technique between the years 2008 to 2022. There were 69 males (84.1%) and 13 females (15.9%). The mean age at the primary operation was 6.1 months. Mean follow-up time was 9.0 years.

**Results:**

There were no major complications related to the procedures. Two patients (2.4%) had a minor dural lesion. There were no postoperative wound infections. Of the 82 patients, seven patients with primary craniosynostosis (13.0%) developed symptomatic craniocerebral disproportion requiring reoperation to increase intracranial volume. In all these patients, invasive intracranial pressure (ICP) monitoring was performed prior to decision-making. In the majority of cases, the aesthetical outcome was considered good or excellent.

**Conclusion:**

The operative method used was feasible and safe. Thirteen percent of patients who were followed over 5 years required major surgery due to development of craniocerebral disproportion later in life.

## Introduction

Craniosynostosis is defined as a condition where one or more cranial sutures are prematurely fused. It is the second most common congenital cause of infant deformity occurring in approximately 1 in 2000 live births [1, 2]. The premature fusion of the skull and facial bones may lead to aesthetical disturbances, malocclusion [3] and psychological disorders [4]. Abnormal head shape may develop depending on which sutures are prematurely fused, the order in which they ossify, and the timing at which it happens. The diagnosis is made by clinical examination along with additional imaging methods like computer tomography (CT) and magnetic resonance imaging (MRI) if required [5]. The premature fusion of the sagittal suture, leading to scaphocephalic head shape, is the most common form of craniosynostosis representing about 40 to 60% of cases [6]. Males have a preponderance in sagittal craniosynostosis [7].

The treatment for craniosynostosis is generally operative. The aim of the surgical treatment is to optimally increase the intracranial volume as well as improving the aesthetics. This can be done by using a variety of methods, including open vault cranioplasty [8], distraction techniques [9], endoscopic strip craniectomies with postoperative moulding therapy with helmets [10] or spring-assisted correction (SAC) [11]. In most cases, the surgery is done during the first year of life [11, 12]. Operative techniques used have been shown to be safe, effective and to have good aesthetical results [8, 13–15]. Surgical complications associated with craniosynostosis surgery are brain injury, blood loss, infections and in rare cases death [16].

Craniosynostosis patients have been reported to have a less pleasing aesthetical appearance [17] and encounter social difficulties related to visible differences in appearance [18, 19]. In many follow-up studies, patients have been reported to have more behavioural disorders in addition to experiencing cognitive and emotional problems [4, 20]. Clinical follow-up is usually continued to school age [15]. Even though this condition has an enormous influence on the patient’s life, only a few studies have had long-term follow-ups through adolescence and adulthood [21].

The aim of this study was to evaluate the incidence of complications and the need for reoperations in nonsyndromic sagittal synostosis patients that were operated on with the H-technique in the primary procedure. Another aim was to evaluate the aesthetical outcomes and the incidence of comorbidities.

## Materials and methods

For this retrospective cohort outcome study, the data on all 111 consecutive patients operated on due to sagittal suture synostosis at the Craniofacial Center of Oulu University Hospital (OUH), Oulu, Finland, between 2008 and 2022 was analysed. The data has been retrospectively collected from medical records and radiological images.

**Fig. 1:**
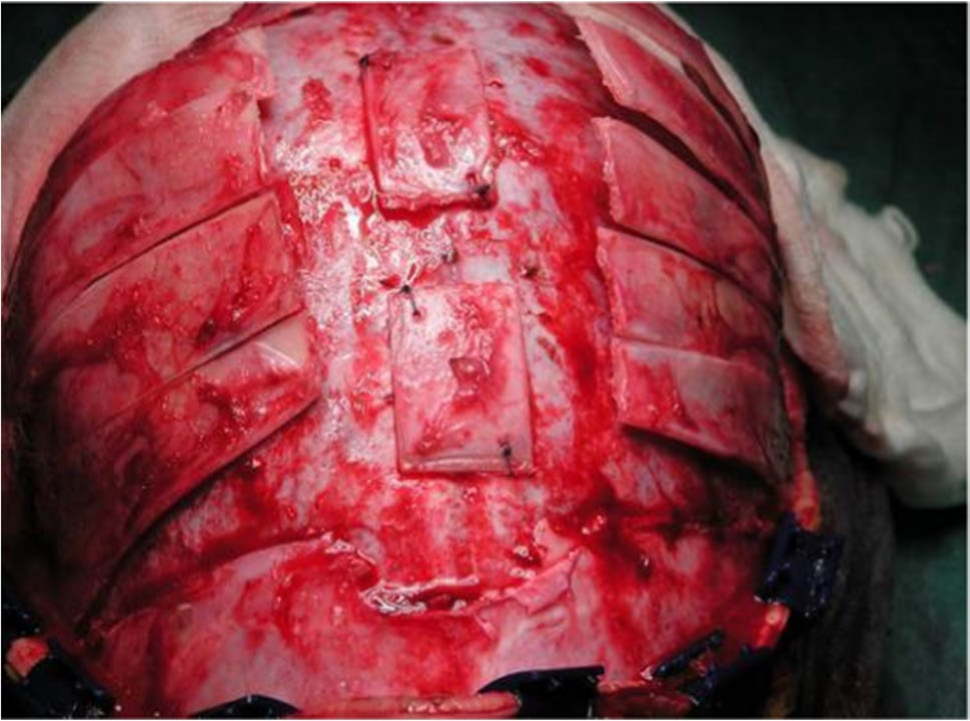
An intraoperative picture of the modified H-technique used in our centre

All patients who appeared to have additionally premature ossification of any other sutures, hydrocephalus or syndromes associated with craniosynostoses diagnosed by the time of the primary surgery were excluded. Thus, the final study group comprised of 82 patients with nonsyndromic sagittal synostosis. All patients were treated using the open vault H-cranioplasty technique with barrel stave osteotomies of the temporal bone that was described earlier [14] (Fig. 1). Additionally, in two patients, the frontal bone was cut, rotated and fixed with sutures to provide better remodelling of the prominent forehead.

According to institutional follow-up protocol, all patients had their first follow-up visits 1–3 months after the operation with further routine annual or biannual follow-up until 7 years of age. Plane lateral and anterior/posterior view skull X-rays were performed at every follow-up for evaluation of ossification of bony defects, rough evaluation of suture status, presence of impressions and follow-up of the skull shape. Clinical examination included evaluation of the aesthetics, skull shape, condition of the scar and palpation of the head. In our institution, there is no routine genetical counselling for typical sagittal synostosis cases.

In all but five cases, the follow-up evaluations were done by one of the craniofacial team members. In the above-mentioned five cases, the evaluation was done by an experienced paediatric surgeon or neurosurgeon in a hospital elsewhere. The families are encouraged to contact the OUH Craniofacial Center directly or through a general practitioner or other medical specialist in case of any possible problems related to craniosynostosis arising later in childhood and adolescence. According to the good practice policy recognised in the whole of Finland, in cases where problems related to the primary surgery or craniosynostoses itself are suspected, the unit where the patient was treated primarily is informed and involved in decision-making even if the patient is treated in another hospital. Thus, whilst the follow-up for aesthetic outcome is ended whenever the routine follow-up is terminated, the follow-up time period for delayed complications and possible development of craniocerebral disproportion is calculated up until the patients’ records were analysed for this study.

When craniocerebral disproportion is suspected, according to Oulu protocol the following studies are performed: plain skull X-ray or a 3D CT scan of the skull, an invasive ICP measurement using the Codman ICP Express ™ monitor for at least 20 h and ophthalmological evaluation of the papilla status. All patients in whom craniocerebral disproportion was suspected were evaluated according to this protocol. The ICP curves were analysed by the principal author (AS) and independently by two seniors (NS and WS).

For evaluation of the aesthetical outcome, a 4-point scale by Aryan [22] was used, as it was done in other studies performed by authors [8, 23] (Table 1).

**Table 1:** 4-point classification of surgical results of craniosynostosis surgery by Aryan et al. (2005)

Score	Description
1	Good to excellent correction. No visible or palpable irregularity
2	Good to excellent correction. Palpable or visible irregularity that does not compromise the overall correction. Does not require repeat operation
3	Compromised correction. Does not require repeat operation
4	Recommended repeat operation either for irregularities or compromised correction

**Fig. 2:**
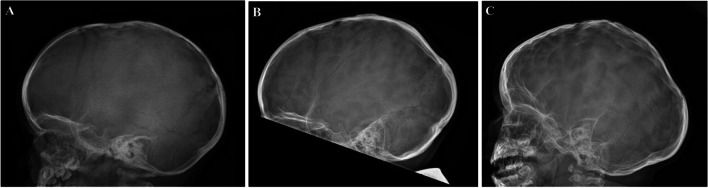
An illustration of the three different impression categories. **A** no impression marks, **B** minor impression marks, normal variation possible and **C** excessive impression marks, suspicion of elevated ICP

For the current study, the quantity of impression marks, “fingerprinting”, was evaluated from the skull X-ray done at the final follow-up visit. Three categories were used: (1) no impression marks, (2) minor impression marks, normal variation possible, (3) excessive impression marks, suspicion of elevated ICP (Fig. 2).

### Ethical board approval

This study was performed according to the principles of the Helsinki declaration. This is a part of a larger study that was approved by the Ethics Review Committee of the Northern Ostrobothnia Hospital District (No. 86/2013). No separate approval for this part of the project was required because this was a retrospective register study with no interference or contact with the patients or follow-up protocols. Therefore, no separate consent was obtained.

### Statistical analyses

All statistical analyses were performed using the IBM SPSS software version 29 (SPSS Inc., Chicago, IL, USA). The Spearman rank correlation test was used for calculating correlations between parameters. *T*-test was used to compare means between the groups. Two-tailed *P* values are presented.

## Results

From the 82 patients included in the study there were 69 males (84.1%) and 13 females (15.9%). At the time of the primary operation the mean age was 6.1 months (range 2.5–18.4 months).

The mean duration of scheduled routine follow-up after the primary operation was 5.2 years (range 11.4 months–14.7 years). In some patients, due to craniocerebral disproportion and reoperations, the follow-up visits continued beyond 7 years of age. At the time the patients’ records were studied, the cohort patients’ mean age was 9.5 years (range 2.2–16.2 years); thus, routine follow-up was finished already in 58 patients. Mean time from the primary operation until the time the patients’ medical documentation was evaluated for the presence of late complications was 9.0 years (range 1.7–15.3 years) (Table 2).

**Table 2:** Patient characteristics

	Value
Age (months) at the time of the primary operation	
Mean	6.1
Range	2.5–18.4
Gender	
Male	69 (84.1%)
Female	13 (15.9%)
Follow-up time for complications (years)	
Mean	9.0
Range	1.8–15.3

There was no mortality (Table 3). Two patients (2.4%) had a minor dural lesion during the operation with no liquorrhea or other wound problems afterwards. There were no postoperative wound infections requiring antibacterial treatment after the primary surgery. Two patients (2.4%) had undergone minor wound revisions 4.7 and 5.5 years after the primary operation due to persisting granulations of the wound.

**Table 3:** Surgical complications

Complication	Value (%)
Mortality	0 (0)
Dural lesion	2 (2.4)
Wound infection/carring	2 (2.4)
Other infection	2 (2.4)
Total	6 (7.3)

*After 5 years from the primary operation due to persisting granuloma of the scar

### Craniocerebral disproportion and reoperations

**Fig. 3:**
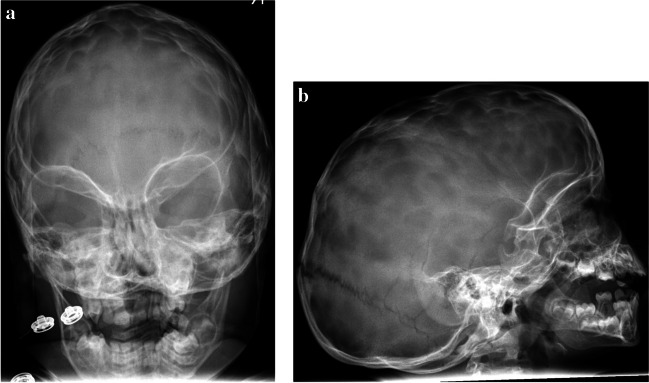
**a** Skull X-ray of patient number 7, frontal view. **b** Skull X-ray of patient number 7, lateral view

Skull X-rays were taken from every patient at the last follow-up visit. Most patients had no (26, 32.9%) or minor impression marks in the X-rays (44, 55.7%). Nine cases had plenty of impressions (11.4%) (Fig. 3a and b) and thus elevated ICP was suspected in these cases. In one of these patients, 3D CT showed an ossified coronal suture on one side with very mild asymmetry, but the 3-year-old patient remained asymptomatic at the time of data analysis. Thus, no ICP measurement has been done to him so far but the follow-up continues. In another patient, the ICP was measured and was found to be normal.

In seven patients, there were complaints related to increased ICP (headache, vomiting, fatigue) and the ICP measurements along with an ophthalmological investigation was performed. ICP was measured invasively using the Codman ICP Express™ monitor for approximately 24 h. The ICP was interpreted as elevated if the value was mainly over 15 mmHg whilst sleeping. In all of these patients, the ICP appeared to be increased and the decision was made to perform the redo surgery in order to increase the intracranial volume. One of these patients (Table 4, Patient 3) was diagnosed with a genetical anomaly (15Q11-13 duplication) soon after the primary operation. This was the only patient who required a third operation by the time of data analysis. In this patient, the ICP was measured only before the third surgery. The decision to perform the first redo surgery was based on clinical symptoms and radiological findings on the 3D CT scan.

**Table 4:** Special characteristic of seven patients with craniocerebral disproportion

Case	Sex	Age at primary operation (mo)	Age at secondary operation (y)	Additional ossified sutures	Operation method	Elevated ICP	Papilledema	Impression marks	Aesthetics	Comorbidities
1	Male	11.9	5.1	Coronal and lambda	PCVD + plates	Yes	No	Major	Excellent	No
2	Male	7.6	4.9	Coronal	PCVD	Yes	No	Major	Excellent	No
3	Female	4.6	1.7	Coronal and lambda	1. PCVD 2. PCVD + plates	Yes	No	Major	Good	Epilepsy, 15Q11-13 *
4	Male	10.1	3.6	Coronal	PCVD + plates	Yes	No	Major	Good	No
5	Male	4.4	3.9	Coronal	PCVD	Yes	Yes	Major	Good	No
6	Male	5.5	1.9	Coronal	PCVD	Yes	No	Major	Good	Headaches**
7	Male	6.0	1.9	Coronal	PCVD	Yes	No	Major	Good	No

*This patient was the only one who required two reoperations due to craniocerebral disproportion

**Explanation in the text, headache unrelated to ICP

From all nine ophthalmological investigations done (eight different patients, from whom 7 required reoperations), only in one patient a papilledema was found.

The status of other sutures was evaluated from 3D CT scans or skull X-rays in patients who needed reoperation. In addition to having a sagittal suture reossification, all patients were found to have developed one or both coronal suture synostosis and two had both coronal and lambda sutures ossified (28.6%). Respectively, for the whole study population, the percentages were 8.7% and 2.5%. Special characteristics of these patients are shown in Table 4. Thus, from all 82 patients, seven cases (8.5%) were diagnosed with craniocerebral disproportion and required major secondary surgery to increase intracranial volume by the time of the data analysis (Table 4).

Age at the reoperation time was 3.3 years (range 1.7–5.1 years) and the time elapsed from the primary surgery was 2.7 years (range 1.4–2.7 years). The posterior cranial vault distraction technique (PVDT) was used in five cases, one stage barrel stave method in one case and in another case a modified PCVD was used, with the bone flap expansion using biodegradable plates. The patient who required two secondary surgeries underwent PCVD and then another PCVD with additional expansion of the bone flap. Six of these patients were males, and the only female patient was the one with the genetical abnormality. By the time of the data analysis, 54 patients had reached 5 years of age. When analysing necessity for reoperations in this group of patients, the risk of developing craniocerebral disproportion increases to 13.0%.

No major complications were present in the reoperation group, though two patients needed revisions related to the distraction devices. In one case due to hardware failure and in another patient due to a wound infection in the distraction device. In one patient, the ICP was measured 13.3 years after the second operation due to headaches after the redo surgery, but the ICP was normal and the headaches were considered unrelated to the craniocerebral disproportion (Patient 6, Table 4).

### Aesthetics

For two out of 82 patients, data on the aesthetical outcome was scarce; the only data available was that the patients were doing well, with no complications neither a need for reoperation for any reasons. One patient was fully lost for follow-up. In most cases, the aesthetical result was either excellent (12, 15.2%) or good (64, 81.0%). Only three cases (3.8%) were classified as having satisfactory, and none as poor aesthetical outcome.

### Comorbidities

The incidence of different comorbidities was assessed from the medical records of the last available follow-up visit (Table 5). The number of patients that had any additional comorbidities diagnosed during follow-up was 15 (19.0%). In this group, 26.7% had two or more comorbidities. Two (2.5%) patients experienced weekly headaches without craniocerebral disproportion. Epilepsy was diagnosed in two cases (2.5%). Speech difficulties and/or behavioural disorders were experienced by four patients (5.1%). In three cases (3.8%), attention-deficit/hyperactivity disorder (ADHD) was diagnosed. One patient (1.3%) had strabismus. Of seven patients with craniocerebral disproportion, two patients had comorbidities. In the comorbidity group (*N* = 15), 13.3% needed secondary surgery due to craniocerebral disproportion.

**Table 5:** Comorbidity and characteristics of each comorbidity

Comorbidity (*N*)	Mean age at operation (months)	Reoperations	ICP measured	Impressions on X-Ray
Headache (2)	4.3	1 (50%)	1 (50%), elevated	1 major, 1 minor
Epilepsy (2)	4.3	1 (50%)	1 (50%), elevated	1 major and 1 minor
Speech and/or behavioural difficulties (4)	5.3	0	0	Minor
ADHD (3)	6.6	0	0	Minor
Strabismus (1)	6.0	0	0	Minor

*Needed one reoperation, ICP was measured twice

**Diagnosed later with 15Q11-13 duplication

Cardiofaciocutaneous syndromes were found in one case and one patient had 15Q11-13 duplication. Hydrocephalus developed in one case and was treated with a ventriculoperitoneal shunt. Later, this patient was diagnosed with a chromosome 5 duplication. One patient was diagnosed with a ventricular septal defect, hydronephrosis, deafness and syndactyly but no known syndrome has been found despite large genetical studies.

## Discussion

This retrospective single-institution study was conducted to evaluate the safety and efficacy of the modified H-technique method used in addition the presence of comorbidities discovered during follow-up was evaluated.

The number of complications in craniosynostosis surgery has dramatically changed during the last decade since the operative methods have evolved [6, 16, 24]. Moreover, the need for reoperations in craniosynostosis varies depending on the type of craniosynostosis, the operation technique used and the age of the patient at the primary operation [11, 24–26]. The current operative methods include modern techniques like Renier’s H-technique [27], endoscopic strip craniectomies with postoperative moulding therapy with helmets [10, 28] or spring-assisted correction (SAC) [11]. Endoscopic and SAC techniques do have shorter operative times and hospital stays in addition to lower blood loss [10, 24]. However, multiple operations are often needed for device placement and removal, or the results depend on using helmets or other assisting devices.

In this study, there were no major complications or mortality. Two patients had delayed minor revisions done due to persisting granulomatous tissue in the scar area years after the primary procedure. In a study conducted by Zakhary et al. using open cranial vault reshaping, the overall complication rate was up to 21%. Though half of the patients in this study had sagittal synostosis, it covered all craniosynostosis types, including syndromic cases [2]. Seruya and associates established a 3.3% complication rate using open cranial vault repair having 45% of the study population consisting of sagittal synostosis patients [26]. These complications included hematomas, cerebrospinal fluid leaks and wound breakdowns. Such complications were not present in our study. Bruce et al. observed an increased risk of perioperative complications in patients who were 12 months or older at the time of primary surgery [29]. In a recently published study [30], blood transfusion was associated with an increased risk of deep surgical site infections in patients with syndromic multi-suture craniosynostosis.

In our study population, seven patients required major surgery due to craniocerebral disproportion resulting in an 8.5% secondary surgery rate. All these patients were symptomatic, had significant impressions on the skull X-rays and had confirmed elevated ICP. According to our institutional policy, the skull CT is not a part of routine protocol. Its necessity is weighted separately in each particular case. In three out of these seven patients, it was not performed, thus decreasing these patients’ ionising radiation load.

All the patients had the craniocerebral disproportion diagnosed by 5 years of age. Therefore, the risk of craniostenosis was evaluated separately for children of at least 5 years of age at the time of data analysis. In this patient cohort (*N* = 54), there was a 13.0% incidence of craniocerebral disproportion. This can be considered the real risk in our population, since the remaining 25 patients were under 5 years of age by the time of data extraction and thus, they still have a possibility of disproportion being revealed on follow-up whilst they grow.

According to previous studies, the reoperation rates due to craniocerebral disproportion vary from 1 to 9% depending on the study population and operative technique used [11, 25, 26, 31]. According to some of the previous studies performing surgery before the age of 6 months, it has been suspected to increase the risk of reoperation and in some cases depending on the operative method used [11, 26]. In our study population, there was no correlation between the age at primary operation and necessity for the redo surgery. However, the size of this patient group was too small to do any reliable statistical calculations. All patients were males and had coronal suture ossification as a new finding (excluding a female who had 15Q11-13 duplication) in our study which raises questions whether further research and genetical investigations are required.

Each patient in our study had symptoms of elevated ICP such as headaches, fatigue and alternations in behaviour, such as irritability, sleeping problems and touching of the head. In a recently published study consisting of 184 nonsyndromic sagittal synostosis patients who were treated with open vault remodelling, elevated ICP was found in 7.7% of the cases and the age at the time of operation of less than 6 months increased this risk [32].

In this study population, papilledema was found only in one patient out of all nine who underwent ophthalmological evaluation due to suspicion of increased ICP. This is a surprising finding, since in seven of these patients, an increased ICP was confirmed. There are only a few studies evaluating the presence of papilledema in nonsyndromic sagittal synostosis patients. Van de Beeten and colleagues found a 6.13% overall incidence of postoperative papilledema of which 70% required reoperation because of craniocerebral disproportion [33] whilst in other studies the incidence varied from 8.9 to 9.7% [34, 35]. In previously mentioned studies, all patients who were included underwent an ophthalmological examination whereas in our population only symptomatic patients were examined. It is important to keep in mind that the absence of papilledema does not ensure normal ICP [36]. In a recently published meta-analysis, no correlation between elevated ICP and negative cognitive outcome was found [37].

In our institution, there is no routine genetic counselling for single-suture sagittal synostosis. Only patients with suspicion of craniofacial syndromes, including all primary coronal suture craniosynostoses, are sent for genetic evaluation. Johnson et al. suggested no mandatory first-line genetic testing for isolated, “typical” sagittal craniosynostosis where there is no family history since studies show a relative low success rate of 6% for molecular testing in these cases [38–40].

The mean age at the secondary surgery was 3.3 years. The second surgery was done mean 2.7 years after the primary surgery. According to the previous publications, the time to redo surgery varies from 2 to 4 years [11, 24, 31, 32]. In our population, all cases of craniocerebral disproportion were diagnosed by 5 years of age. None required reoperations due to purely aesthetical reasons. Thus, it can be speculated that the risk of craniocerebral disproportion development is low after 5 years of age and thereby follow-up until school age is sufficient. All seven patients with craniocerebral disproportion had sagittal and coronal suture ossification, and two patients had additionally lambda suture premature ossification, therefore exhibiting progression from single-suture craniosynostosis to multiple suture craniosynostosis. Similarly, Arnaud et al. found that secondary coronal synostosis was present in 10% of the cases and 1% requiring surgical intervention.

The aesthetical result was excellent or good in the majority of cases (96.2%). None of the patients required redo surgery due to aesthetical reasons. This finding is in line with the previous findings that the aesthetical result is good in operatively treated patients who along with their caregivers are satisfied with their appearance [14, 15, 26, 41]. A good aesthetical outcome and thus satisfaction can have a major impact on self-esteem and therefore on quality of life [42, 43].

Sagittal craniosynostosis patients have been shown to have up to 30–39% risk of speech and language difficulties [4, 44]. In our study, 5.1% of the patients had delayed speech and/or language development resulting in a necessity for additional support in preschool. None of these patients had symptoms or signs of elevated ICP and thus had no ICP monitoring done. ADHD has been shown to have an overrepresentation in craniosynostosis patients when comparing with normal population [45]. In a recently published meta-analysis, sagittal synostosis patients were found to have more attention, long-term memory and visuospatial ability problems [46]. However, the sample sizes of these studies were small and the findings disperse. Nevertheless, sagittal synostosis patients have been shown to have as good socioeconomic situation, general health and psychological status as controls [14, 43]. In this study, the comorbidities were evaluated objectively from medical records whilst in the previously published study, patients’ self-reported data was used, leaving a possibility for bias [14].

Epilepsy requiring medication was diagnosed in two cases of our patients (2.5%) during the follow-up period. In one of these cases, a genetical abnormality (duplication of 15Q11-13) was found. Whilst studies of the incidence of epilepsy in nonsyndromic scaphocephaly patients are lacking, hydrocephalus and brain compression have been shown to increase the risk of epilepsy in craniosynostosis patients [47]. Rajkumar et al. found a 1.32% seizure and 0.43% cerebral palsy incidence in their study population [30].

### Strengths and limitations

Using different classifications of complications, different surgical techniques and different follow-up times and having heterogenous study populations make it difficult to compare results of different studies with each other.

The weakness of the present study is that for some patients, the follow-up period was short, being under 2 years in one case and under 3 years in six cases, thus making data on development of craniocerebral disproportion, comorbidities and delayed complications unreliable for them. Additionally, we are still unable to determine the risk factors for the development of craniocerebral disproportion and need for reoperations.

The strength of this study is that all operations were done by the members of the Oulu Craniofacial team using the same operative technique. The patients are rarely lost for follow-up in Finland and having national policy of informing the centre where the primary surgery was done about all possible further interventions improves the quality of the data.

## Conclusion

The modified H-technique is a safe and feasible surgical method in the treatment of sagittal suture synostosis. It provides good aesthetical results. The incidence of craniocerebral disproportion requiring interventions was found to be 13.0% in when follow-up was sufficiently long. Necessity for secondary surgery appeared by 5 years of age, confirming that the existing protocol of follow-up until 7 years of age is sufficient to detect all these cases. The finding that majority of patients who needed reoperations were males and had additional coronal synostosis is interesting and should be a topic of future research.

## Data Availability

This manuscript has no associated data.
